# Single nucleotide polymorphisms, haplotypes and combined genotypes in *MYH3* gene and their associations with growth and carcass traits in Qinchuan cattle

**DOI:** 10.1007/s11033-012-2076-z

**Published:** 2012-10-17

**Authors:** Lijun Wang, Xiaolin Liu, Fubiao Niu, Hongliang Wang, Hua He, Yulan Gu

**Affiliations:** College of Animal Science and Technology, Northwest A&F University, Yangling, 712100 Shaanxi People’s Republic of China

**Keywords:** Qinchuan cattle, Growth and carcass traits, Combined genotypes, Haplotype, *MYH*_*3*_

## Abstract

*MYH*_*3*_ is a major contractile protein which converts chemical energy into mechanical energy through the ATP hydrolysis. *MYH*_*3*_ is mainly expressed in the skeletal muscle in different stages especially embryonic period, and it has a role in the development of skeletal muscle and heart. In this study, polymerase chain reaction-restriction fragment length polymorphism (PCR-RFLP) was applied to analyze the genetic variations of the *MYH*_*3*_ gene and verify the effect on growth and carcass traits in a total of 365 Qinchuan cattles. The PCR product was digested with some restriction enzyme and demonstrated the polymorphism in the population, the single nucleotide polymorphisms (SNPs) at nucleotides g. +1215T>C, g. +3377C>T, and g. +28625C>T were in linkage disequilibrium with each other. The result of haplotype analysis showed that nineteen different haplotypes were identified among the five SNPs. The statistical analyses indicated that the five SNPs were significant association with growth and carcass traits (*P* < 0.05, *N* = 365); whereas the five SNPs were no significant association between 18 combined genotypes of *MYH*_*3*_ gene and growth and carcass traits. Taken together, our results provide the evidence that polymorphisms in *MYH*_*3*_ are associated with growth and carcass traits in Qinchuan cattle, and may be used as a possible candidate for marker-assisted selection and management in beef cattle breeding program.

## Introduction

Multiple myosin isoforms are encoded by a multigene family and expressed in different developmental stages and fiber types. There are three skeletal fast myosin heavy chain (*MyHC*) genes (IIa, IIdx, and IIb), but only the promoter region of the mouse IIb gene has been analyzed to date [1–6]. The *MyHCs* encoded by a group of genes consisting of IIa, IIb [7], IIx [8], extraocular [9], embryonic [10] and neonatal [11] genes. These genes are located on the chromosome 14 [12], which expressed at different times during development and in different fiber types [13–16].

Myosin proteins consist of both heavy and light chains and are present in skeletal muscle. Heavy chains are associated with the speed of muscular contractions, while the role of light chains is not well defined [17]. Studies have shown that myosin heavy chain 3 (*MYH*_*3*_) gene may be one of major genes implicated in the differences of muscle fiber property between intact males and castrated Qinchuan cattle [18]. The *MYH*_*3*_ is a major structural protein of the thick filament of the sarcomere. *MYH*_*3*_ gene is a member of the *MYH* family and encodes a protein with an IQ domain [19]. Polymorphism of *MHC* isoform expression in single myofibers, which points out that single fibers of both developing and adult skeletal muscle exist as hybrids [20–23]. In a word, *MYH*_*3*_ gene is expressed during the embryonic period [24], at the same time, *MYH*_*3*_ has a role in skeletal muscle development [13, 25, 26].

In human, the *MYH*_*3*_ gene is located on chromosome 17, and it is a molecular motor that converts chemical energy into mechanical force [27]. Conventionally, class II myosin is a hexameric protein composed of two *MyHC* subunits, each with a molecular weight of approximately 220 kDa and two pairs of non-identical myosin light chain subunits. Proteolytic enzymes can cleave the *MyHC* into two sub-fragments: heavy meromyosin (*HMM*) and light meromyosin (*LMM*). The expression of *MYH*_*3*_ is predominated in myotubes fated to become fast myofibers and is gradually replaced by the expressions of other myosin genes (*MYH*_*1*_, *MYH*_*2*_ and *MYH*_*4*_) [28]. During the development and the adult, *α*-*MYH* is predominately expressed in the atrial chamber and *β*-*MYH* in the ventricular chamber in both humans and chicks [29–33]. This is further illustrated by the increased expression of *β*-*MHC* in the atria of human hearts under pressure overload [34, 35]. *MHC* analysis of human soleus muscle shows that this muscle expresses an approximately equal mix of type I and IIa isoforms, but the type IIx *MHC* is not expressed [36]. In contrast, human fast muscles such as the vastus lateralis express a mix of all three types of *MHC* isoforms at variable proportions [36, 37], depending on the physical fitness and activity of the subject. In addition, the study shows that knockdown in the early embryo leads to abnormal atrial septal development and heart enlargement [38]. These studies all point to the potentially vital function of *MYH*_*3*_ in regulating gene expression at the skeletal muscle and heart development.

In a rat model, the *MYH*_*3*_ gene is located on chromosome 10. It is an essential protein with skeletal muscle development. *β*-*MHC* has been shown to be down-regulated in the setting of hypoxia, likely as a result of neuroendocrine stimulation [39]. Cytokines and tumor factors such as *TNF*-*α* have been demonstrated to selectively target *MYH*_*2*_ gene and down-regulate transcription in a mouse model [40]. *MYH*_*3*_ is plastic in the adult rodent heart. In contrast to described human, the adult heart of small animals such as rodents and mice mainly expresses the *α*-*MHC* throughout the adult state. These results also support the idea that *MYH*_*3*_ plays an important role in heart development.

In this study, we used DNA sequencing, PCR-RFLP analysis to investigate allelic variation of *MYH*_*3*_ gene that encompasses five SNPs. Furthermore, we reported associations among these SNPs of the *MYH*_*3*_ gene in the Qinchuan cattle breed and the linkage disequilibrium between the variations and we analyzed the relationship between single nucleotide polymorphisms and growth and carcass traits.

## Materials and methods

### Animal source and preparation of DNA samples

Cattle from the Qinchuan (QC) commercial breeds were randomly selected, for a total of 365 animals. These animals (30 ± 2 months of the age at slaughter) were reared in Shaanxi province, P.R. China. The growth traits (body length, withers height and hip width) and carcass traits (slaughter weight and carcass weight) were measured according to the criteria of GB/T17238-1998 Cutting Standard of Fresh and Chilled Beef in China (China Standard Publishing House). All experimental protocols and animal care were performed according to authorization granted by the Chinese Ministry of Agriculture. Genomic DNA was isolated from 2 % heparin-treated blood samples and stored at −80 °C, following the standard procedures [41].

### PCR amplification and DNA sequencing analysis

Primers used to amplify bovine *MYH*_*3*_ gene were designed from a published gene sequence (GenBank accession number NC_007317). Primers, restriction enzymes selected (ABI, Foster City CA), The primer sequences, location and fragment sizes were listed in Table 1. The detection of allelic variation at the SNPs were based on the electrophoretic pattern of the restriction enzyme-treated PCR products.

**Table 1:** Primer sets for PCR and PCR-RFLP used for genotyping SNPs detected in bovine *MYH*_*3*_ gene

SNP_S_	Amplified region	Primer sequence (5′–3′)	AT(°C)	SAF (bp)	PR	Genotype
SNP1	Exon5	AAAGCCCGAGTATCAGAACC TCCAACAGCCTCATCAAACA	57.4	435	*RSa*I	CC: 228, 207
						CT:228, 207, 150, 78
						TT:207, 150, 78
SNP2	Intron5	AAAGCCCGAGTATCAGAACC TCCAACAGCCTCATCAAACA	57.4	435	*Bst*XI	TT:435
						TC:435, 339, 96
						CC: 339, 96
SNP3	Intron7	TTGCTGCAAATGGCATTATT TGAGATTCCCAACAAAGAGG	59.8	447	*PVU*II	GG:447
						GC:447, 222, 225
						CC: 222, 225
SNP4	Exon14	TGGAGGATTTTCAGAGGGGT TGATATGGGGTGACAAGTGG	59.3	532	*RSa*I	CC:532
						CT:532, 364, 168
						TT:364, 168
SNP5	Exon14	TGGAGGATTTTCAGAGGGGT TGATATGGGGTGACAAGTGG	59.3	532	*Taq*I	CC:433, 99
						CT:433, 243, 190, 99
						TT:243, 190, 99

SNPs: single nucleotide polymorphisms; SNP1 = C1878T; SNP2 = T2010C; SNP3 = G3746C; SNP4 = C7294T; SNP5 = C7315T

AT: annealing temperature, SAF: size of amplification fragment, PR: PCR-RFLP

PCR amplifications were performed in a total volume of 15 μL, where the volume mixture contained: 50 ng of genomic DNA as template, 10 mM Tris–HCl buffer (pH 8.8), containing 50 mM KCl, 0.2 μM of each primer, 200 μM dNTP and 0.5 U Taq DNA polymerase (MBI Fermentas, USA). The Mg^2+^ concentration was optimized for each primer set. PCR conditions were as follows: after an initial denaturation of 5 min at 95 °C, amplicons were generated for 35 cycles of 30 s at 94 °C, 30 s at an optimal annealing temperature, and 45 s at 72 °C, followed by a 10 min final extension at 72 °C.

Restriction fragment length polymorphism analysis was used to identify the genotypes of sequence variants. The PCR products were digested in a total volume of 10 μL containing: 5 μL of PCR product, three units of restriction enzyme (0.3 μL), 1 μL of reaction buffer and 3.7 μL of ddH_2_O. The mixture was incubated for 10 h at specific temperature (*Rsa*I, *Bst*XI, *PVU*II and *Taq*I restriction enzymes were digested at 37, 55, 37 and 65 °C, respectively). Digested PCR products were mixed with 10× loading buffer and subjected to 2.5 % agarose gel electrophoresis in 1× TBE at constant voltage (110 V) for 1.0 h at room temperature. Individuals were then genotyped based on different electrophoresis patterns. Fragments displaying different PCR-RFLP patterns were purified with Qiaquick spin columns (Qiagen) and sequenced with the ABI PRISM 3730 sequencer (ABI, Foster City CA) and sequences were analyzed with BioXM software (Version 2.6). The sequences obtained were named with letters of the alphabet.

### Linkage disequilibrium and statistical analysis

1. Genotypic frequency and allelic frequency were determined for each breed by direct counting. The formulas were as follows:

$$ {\text{GF}}_{\text{i}} = {\text{n}}_{\text{i}} /{\text{N AF}}_{\text{i}} = 2 {\text{Nii}} + {\text{Nij}}/ 2 {\text{N}} $$

(n_i_ is the number of i genotypic, “N_i_” is the frequency of the i allele, “N” is the number of alleles)

(2) The pattern of pairwise linkage disequilibrium (LD) between the SNPs was measured by LD coefficient (D′) and correlation coefficient (*r* ^2^), the measurements were determined using the program Haploview (http://www.broad.mit.edu/mpg/haploview) [42].
(3) The χ^*2*^ test was used to determine Hardy–Weinberg equilibrium of the mutation. Population genetic indices, including heterozygosity (*He*), homozygosity (*Ho*), polymorphism information content (*PIC*) and effective allele numbers (*Ne*) were calculated according to Nei et al. [43]. The formulas were as follows:

$$ H_{o} = \sum\limits_{i = 1}^{n} {P_{i}^{2} } H_{e} = 1 - \sum\limits_{i = 1}^{n} {P_{i}^{2} } Ne = {1 \mathord{\left/ {\vphantom {1 {\sum\limits_{i = 1}^{n} {P_{i}^{2} } }}} \right. \kern-0pt} {\sum\limits_{i = 1}^{n} {P_{i}^{2} } }}PIC = 1 - \sum\limits_{i = 1}^{m} {P_{i}^{2} } - \sum\limits_{i = 1}^{m - 1} {\sum\limits_{j = i + 1}^{m} {2P_{i}^{2} P_{j}^{2} } } $$

(“P_i_” is the frequency of the i allele, “n” is the number of alleles)

(4) Haplotypes were obtained for each animal using the PHASE computer program (Ver 2.1) [44].
(5) The SPSS software (Version 16.0) was used to analyze the association between genotypes and traits in Qinchuan cattle.
(6) Combined genotypes of SNPs and growth traits association analyses were carried out to explore the possible interaction between the SNPs. The model was similar to that of single marker association analysis, except that the interaction between the two SVs was included as a fixed effect.

## Results

### Sequence variants identified of *MYH*_*3*_ gene

The bovine *MYH*_*3*_ gene located on chromosome 19, and has been revealed that it contained 42 exons and encoded 1249 amino acids. In the present study, Genomic DNA of Qinchuan breeds was successfully amplified using primer pairs for the *MYH*_*3*_ gene (Table 1).

In total, five variants including three exons and two introns mutation were identified in the study (Table 2). According to the information of cattle *MYH*_*3*_ (GenBank Accession number: NC_007317), the T2010C and G3746C mutations were in intron 5 and intron 7, respectively, while the C1878T, C7294T and C7315T mutations were in exon 5, exon 14 and exon 14, respectively. According to the sequence mutations, the PCR products could be digested with *RSa*I, *Bst*XI, *PVU*II and *Taq*I restriction enzymes. Distinct banding patterns were analyzed in this study and shown in Table 2. The SNPs of g. +1878C>T and g. +7315C>T resulted in synonymous mutations of Tyr626Tyr and Val2439Val, respectively, while the SNP of g. +7294C>T brought a missense mutation Arg2432Cys. Interestingly, it was firstly found that five novel mutations could be detected by endonucleases restriction site in bovine *MYH*_*3*_ gene.

**Table 2:** Description of the SNPs at the bovine *MYH*_*3*_ gene

SNPs name	Alleles	Amino acid change
SNP1 C1878T	TAC/TAT	Tyr626Tyr
SNP2 T2010C	TGT/TGC	–
SNP3 G3746C	TGT/TCT	–
SNP4 C7294T	CGT/TGT	Arg2432Cys
SNP5 C7315T	CGA/TGA	Val2439Val

*Note*: SNPs: single nucleotide polymorphisms; SNP1 = C1878T; SNP2 = T2010C; SNP3 = G3746C; SNP4 = C7294T; SNP5 = C7315T

It was a very useful strategy to scan large sample size sequence mutations with the methods of DNA sequencing and PCR-RFLP, which would overcome the inaccuracy, the complicated technical demands, slow speed and unstable reproducibility. Interestingly, in this study, the PCR-RFLP method was successfully carried out to accurately detect the polymorphisms of the *MYH*_*3*_ gene.

**Fig. 1:**
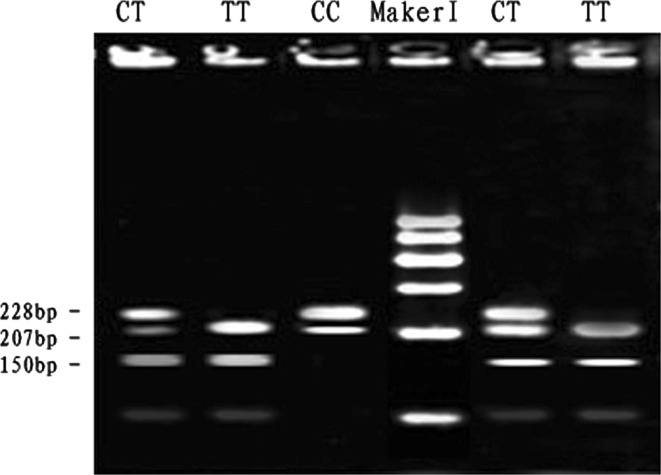
The 3 % agarose gels electrophoretic patterns of the *MYH*_*3*_ gene in bovine. M = DNA molecular weight marker is Marker I; C1878T genotype: CC = 228 + 207 bp, TT = 207 + 150 + 78 bp, CT = 228 + 207 + 150 + 78 bp; It is difficult to see the 78 bp DNA fragment on 3 % agarose gel. Forced PCR-RFLP detection results of *MYH*_*3*_ gene PCR product

At the SNP1-*RSa*I locus, digestion of the 435 bp PCR fragment of *MYH*_*3*_ exon 5 with *RSa*I resulted in fragment lengths of 228, 207, 150 and 78 bp for genotype CT; 228, 207, 150 and 78 bp for genotype TT and 228 and 207 bp for genotype CC (see Fig. 1). The frequencies of genotype and allele were calculated in the Qinchuan bovine population (Table 2). The frequency of allele T was dominant in the Qinchuan cattles and CT genotype was more frequent than other genotypes. The genotypic frequencies of SNP1-*RSa*I locus in Qinchuan cattle population agreed with Hardy–Weinberg disequilibrium (0.01 < *P* < 0.05) (Table 3).

**Table 3:** Genotypic and allelic frequencies (%), value of χ2 test and diversity parameter of bovine *MYH*_*3*_ gene

SNPs	Genotype	Number	GF	Allele	AF	χ^2^(HWE)^e^	*He*	*H*_*O*_	*Ne*	*PIC*
SNP1	CC	51	0.1397	C	0.4205					
	CT	205	0.5616	T	0.5795	8.4758	0.4874	0.5126	1.9508	0.3686
	TT	109	0.2986							
SNP2	TT	56	0.1534	T	0.4233					
	TC	197	0.5397	C	0.5767	4.0605	0.4882	0.5118	1.9539	0.3690
	CC	112	0.3068							
SNP3	GG	43	0.1178	G	0.4384					
	GC	234	0.6411	C	0.5616	33.2854	0.4924	0.5076	1.9701	0.3712
	CC	88	0.2411							
SNP4	CC	39	0.1068	C	0.4068					
	CT	219	0.6000	T	0.5932	21.5790	0.4826	0.5174	1.9327	0.3661
	TT	107	0.2932							
SNP5	CC	46	0.1260	C	0.4123					
	CT	209	0.5726	T	0.5877	12.0281	0.4846	0.5154	1.9402	0.3672
	TT	110	0.3014							

*Note*: SNPs: single nucleotide polymorphisms; SNP1 = C1878T; SNP2 = T2010C; SNP3 = G3746C; SNP4 = C7294T; SNP5 = C7315T

Number: genotype number

GF: genotypic frequency; AF: allelic frequency; *χ* ^*2*^(HWE): Hardy–Weinberg equilibrium χ^2^ value; He: gene heterozygosity; Ho: gene homozygosity; Ne: effective allele numbers; PIC: polymorphism information content

**Fig. 2:**
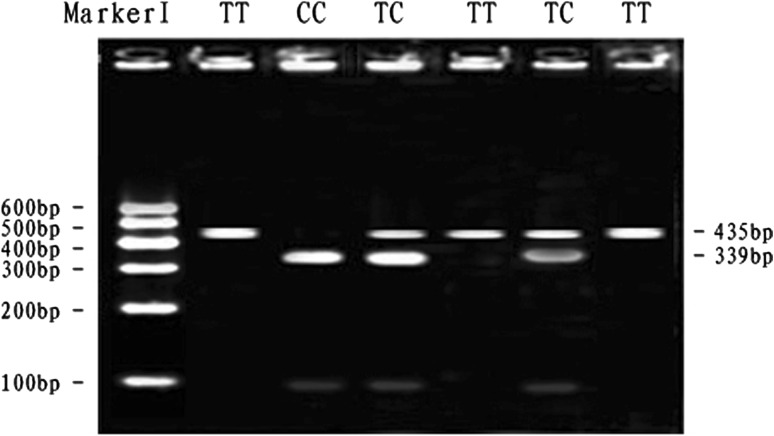
The 3 % agarose gels electrophoretic patterns of the *MYH*_*3*_ gene in bovine. M = DNA molecular weight marker is Marker I; T2010C genotype: TT = 435 bp, TC = 435 + 339 + 96 bp, CC = 339 + 96 bp; It is difficult to see the 96 bp DNA fragment on 3 % agarose gel

At the SNP2-*Bst*XI locus, digestion of the 435 bp PCR fragment with *Bst*XI resulted in fragment lengths of 435 bp for genotype TT; 435, 339 and 96 bp for genotype TC and 339 and 96 bp for genotype CC (see Fig. 2). The frequency of allele C was dominant in the Qinchuan breeds and TC genotype was more frequent than other genotypes. The genotypic frequencies of SNP2-*Bst*XI locus in Qinchuan cattle population agreed with Hardy–Weinberg equilibrium (*P* > 0.05) (Table 3).

**Fig. 3:**
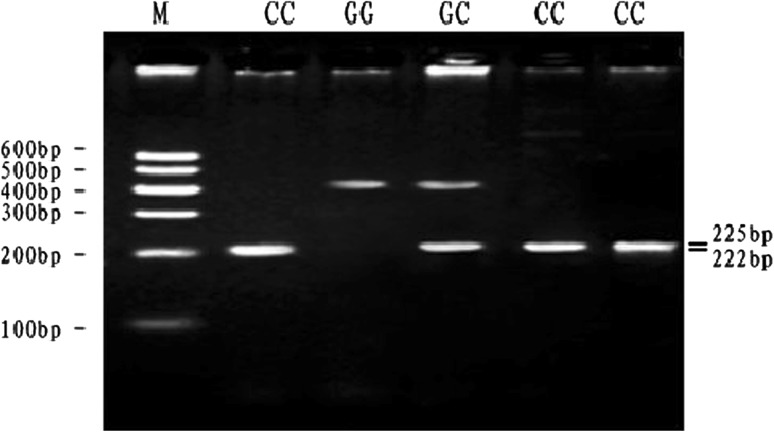
The 2.5 % agarose gels electrophoretic patterns of the *MYH*_*3*_ gene in bovine. M = DNA molecular weight marker is Marker I; G3746C genotype: GG = 447 bp, GC = 447 + 225 + 222 bp, CC = 225 + 222 bp; It is difficult to see the 222 bp and 225 bp DNA fragment on 2.5 % agarose gel

At the SNP3-*PVU*II locus, digestion of the 447 bp PCR fragment with *PVU*II resulted in fragment lengths of 447 bp for genotype GG; 447, 225 and 222 bp for genotype GC and 225 and 222 bp for genotype CC (see Fig. 3). The frequency of allele C was dominant in the Qinchuan breeds and GC genotype was more frequent than other genotypes. The genotypic frequencies of SNP3-*PVU*II locus in Qinchuan cattle population agreed with Hardy–Weinberg disequilibrium (*P* < 0.01) (Table 3).

**Fig. 4:**
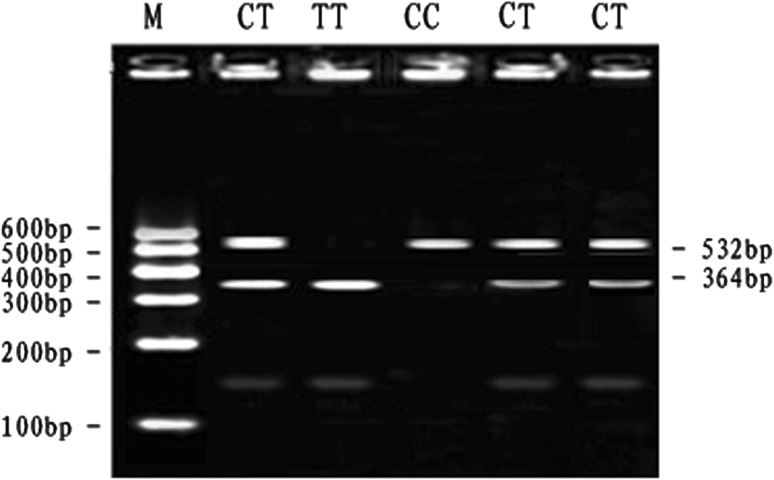
The 2.5 % agarose gels electrophoretic patterns of the *MYH*_*3*_ gene in bovine. M = DNA molecular weight marker is Marker I; C7294T genotype: CC = 532 bp, CT = 532 + 364 + 168 bp, TT = 364 + 168 bp; It is difficult to see the 168 bp DNA fragment on 2.5 % agarose gel

At the SNP4-*RSa*I locus, digestion of the 532 bp PCR fragment with *RSa*I resulted in fragment lengths of 532 bp for genotype CC; 532, 364 and 168 bp for genotype CT and 364 and 168 bp for genotype TT (see Fig. 4). The frequency of allele T was dominant in the Qinchuan breeds and CT genotype was more frequent than other genotypes. The genotypic frequencies of SNP4-*RSa*I locus in Qinchuan cattle population agreed with Hardy–Weinberg disequilibrium (*P* < 0.01) (Table 3).

**Fig. 5:**
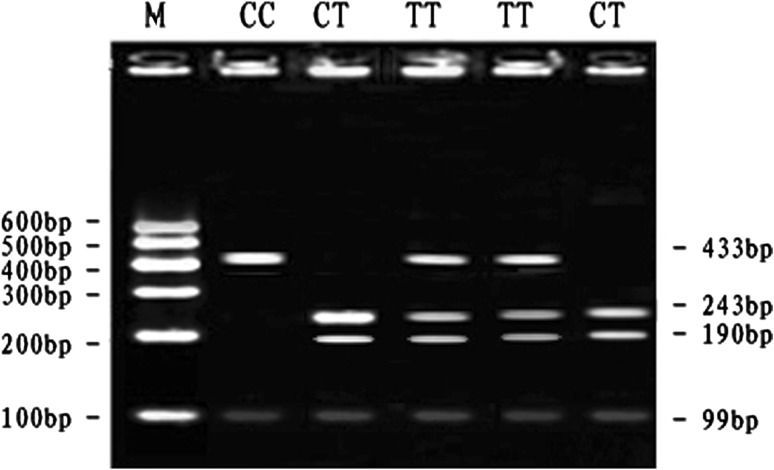
The 3 % agarose gels electrophoretic patterns of the *MYH*_*3*_ gene in bovine. M = DNA molecular weight marker is MarkerI; C7315T genotype: CC = 433 + 99 bp, CT = 243 + 190 + 99 bp, TT = 433 + 243 + 190 + 99 bp

At the SNP5-*Taq*I locus, digestion of the 433 bp PCR fragment with *Taq*I resulted in fragment lengths of 433 bp for genotype CC; 433, 243 and 190 bp for genotype CT and 243 and 190 bp for genotype TT (see Fig. 5). The frequency of allele T was dominant in the Qinchuan and CT genotype was more frequent than other genotypes. The genotypic frequencies of SNP5-*Taq*I locus in Qinchuan cattle population agreed with Hardy–Weinberg disequilibrium (*P* < 0.01) (Table 3).

### Analysis of polymorphism of the *MYH*_*3*_ in Qinchuan cattle population

The means and standard deviations (SD) for traits were analyzed in this study shown in Table 4. The allelic and genotypic frequencies, genetic diversity parameters (*Ho*, *He*, *Ne* and *PIC*) of the five SNPs were shown in Table 3. According to Table 3, only one loci of Qinchuan cattle was in Hardy–Weinberg equilibrium (*P* > 0.05). It showed that the Qinchuan cattle breed was in a dynamic disequilibrium in artificial selection, migration and genetic drift function. The maximum and minimum *PIC* values were 0.3661 and 0.3690. According to the genetic diversity classification of *PIC* (*PIC* value <0.25, low polymorphism; 0.25 < *PIC* value < 0.5, intermediate polymorphism and *PIC* value >0.5, high polymorphism), at the five SNPs, the result reflected that an intermediate genetic diversity of Qinchuan bovine *MYH*_*3*_ gene in the population analyzed.

**Table 4:** Means and standard deviations (SD) for traits

Traits	Mean	SD
Body height (cm)	140.22	4.26
Withers length (cm)	150.71	6.14
Hip width (cm)	48.00	3.66
Slaughter weight (kg)	497.96	63.67
Carcass weight (kg)	268.87	36.99

### Linkage disequilibrium and haplotype analysis of the *MYH*_*3*_ in Qinchuan cattle population

Linkage disequilibrium and haplotype analysis of the *MYH*_*3*_ gene in Qinchuan cattle population were shown in Tables 5 and 6. The linkage disequilibrium between the five SNPs in the population was estimated, which indicated that the *D*′ values ranged from 0.011 to 0.58; the *r* ^2^ values were from 0.000 to 0.292. Moreover, SNP1 and SNP2–SNP5 had little linkage equilibrium (*D*′ < 0.07 and *r* ^2^ < 0.003). SNP2 and SNP3–SNP5 had little linkage equilibrium (*D*′ < 0.08 and *r* ^2^ < 0.003).

**Table 5:** The estimated values of linkage equilibrium analysis between 5 mutation sites within *MYH*_*3*_ gene of studied population

SNPs	1	2	3	4	5
1		D′ = 0.077	D′ = 0.080	D′ = 0.026	D′ = 0.011
2	*r* ^2^ = 0.003		D′ = 0.087	D′ = 0.080	D′ = 0.110
3	*r* ^2^ = 0.002	*r* ^2^ = 0.003		D′ = 0.439	D′ = 0.580
4	*r* ^2^ = 0.001	*r* ^2^ = 0.004	*r* ^2^ = 0.110		D′ = 0.422
5	*r* ^2^ = 0.000	*r* ^2^ = 0.005	*r* ^2^ = 0.292	*r* ^2^ = 0.117	

*SNPs* single nucleotide polymorphisms; 1 = C1878T; 2 = T2010C; 3 = G3746C; 4 = C7294T; 5 = C7315T

D′ and *r* ^2^ above and below the diagonal, respectively

**Table 6:** Haplotype and haplotype frequency within studied population of 3 SNPs in bovine *MYH*_*3*_ gene

Haplotype	SNPs			Frequency in population	Cumulative frequency
	G3746C	C7294T	C7315T		
Hap 1	C	C	C	0.080	0.080
Hap 2	C	C	T	0.149	0.229
Hap 3	C	T	C	0.046	0.275
Hap 4	C	T	T	0.393	0.668
Hap 5	G	C	C	0.210	0.878
Hap 6	G	C	T	0.038	0.916
Hap 7	G	T	C	0.034	0.950
Hap 8	G	T	T	0.050	1.000

The haplotype analysis showed that eight different haplotypes were identified among the three SNPs. Three major haplotypes accounting for 75.2 % of the alleles were obtained as follows, haplotype 2: –CCT– (14.9 %), haplotype 4, –CTT– (39.3 %) and haplotype 5, –GCC– (21.0 %).

### Association analysis of single markers and combined genotypes

The results of the association analyses between five mutations in *MYH*_*3*_ and growth and carcass traits were shown in Table 7. Growth and carcass traits were associated by the analysis of Qinchuan cattle at 30 ± 2 months old. According to Table 7, at locus C1878T, the animals with the CT genotype had greater withers height and heavier slaughter weight than those with the TT genotype (*P* < 0.05). At locus T2010C, the TT genotype was better than the TC and CC genotypes, and the animals with the TT genotype had greater withers height than those with the CC genotype (*P* < 0.05), the animals with the TT genotype had heavier slaughter weight than those with the TC and CC genotypes (*P* < 0.05). At locus G3746C, the animals with the GG genotype had greater body length and withers height than those with the GC and CC genotypes (*P* < 0.05), and GG genotype had greater hip width than those with GC genotype (*P* < 0.05). At locus C7315T, the animals with the CC genotype had heavier slaughter weight than those with CT genotype (*P* < 0.05). The rest of the records of growth and carcass traits had no significant association (*P* > 0.05).

**Table 7:** Association of SNPs with growth and carcass traits

Locus	Genotype	N	Body length (cm) (mean ± SE)	Withers height (cm) (mean ± SE)	Hip width (cm) (mean ± SE)	Slaughter weight (kg) (mean ± SE)	Carcass weight (kg) (mean ± SE)
C1878T	CC	51	140.681 ± 0.882	151.306 ± 0.991^ab^	47.292 ± 0.622	497.056 ± 10.171^ab^	265.425 ± 5.656
	CT	205	140.382 ± 0.524	150.382 ± 0.589^b^	46.750 ± 0.370	495.824 ± 6.043^b^	266.104 ± 3.360
	TT	109	140.753 ± 0.603	152.506 ± 0.678^a^	47.299 ± 0.425	505.623 ± 6.955^a^	273.209 ± 3.868
	P		0.889	0.063	0.564	0.049	0.321
T2010C	TT	56	140.568 ± 0.777	149.676 ± 0.947^b^	46.676 ± 0.573	487.243 ± 9.266	258.881 ± 5.260^b^
	TC	197	140.055 ± 0.637	150.855 ± 0.777^ab^	47.491 ± 0.470	497.036 ± 7.600	268.102 ± 4.314^a^
	CC	112	141.206 ± 0.811	152.735 ± 0.988^a^	46.941 ± 0.598	509.706 ± 9.666	265.806 ± 5.487^a^
	P		0.536	0.043	0.521	0.248	0.032
G3746C	GG	43	138.629 ± 0.940^b^	148.742 ± 1.065^b^	45.758 ± 0.666^b^	484.290 ± 10.908	264.461 ± 6.115
	GC	234	140.964 ± 0.575^a^	151.892 ± 0.651^a^	47.301 ± 0.407^a^	507.000 ± 6.667	271.022 ± 3.737
	CC	88	140.832 ± 0.521^a^	151.594 ± 0.590^a^	47.213 ± 0.369^ab^	498.089 ± 6.043	267.742 ± 3.388
	P		0.085	0.035	0.117	0.198	0.625
C7294T	CC	39	140.353 ± 0.680	151.138 ± 0.782	46.914 ± 0.494	492.552 ± 7.986	266.414 ± 4.518
	CT	219	140.146 ± 0.572	150.524 ± 0.658	47.067 ± 0.416	500.061 ± 6.716	271.487 ± 3.800
	TT	107	141.308 ± 0.643	152.123 ± 0.739	47.146 ± 0.467	501.539 ± 7.544	267.275 ± 4.268
	P		0.376	0.272	0.942	0.681	0.637
C7315T	CC	46	140.283 ± 0.964	149.700 ± 1.094	46.467 ± 0.681	485.100 ± 11.141^b^	268.123 ± 6.243
	CT	209	140.112 ± 0.533	151.837 ± 0.605	47.026 ± 0.377	504.102 ± 6.164^a^	268.125 ± 3.454
	TT	110	141.14 ± 0.569	151.198 ± 0.646	47.192 ± 0.402	499.372 ± 6.580^ab^	269.176 ± 3.687
	P		0.404	0.231	0.657	0.030	0.976

*Note*: Values with different superscripts within the same column differ significantly at *P* < 0.05 (a, b)

*SE* standard error

S

The combined genotypes association results for SNP3, SNP4 and SNP5 were shown in Table 8. The associated analysis suggested that no significant differences were detected between the combined genotypes of three SNPs and growth and carcass traits in Qinchuan cattle (*P* > 0.05).

**Table 8:** Associations between combined genotypes of three SNPs and growth and carcass traits in Qinchuan cattle (N = 365): Mean ± SE

Genotype of combination (19)	Number of combination	Body length (cm) (mean ± SE)	Withers height (cm) (mean ± SE)	Hip width (cm) (mean ± SE)	Slaughter weight (kg) (mean ± SE)	Carcass weight (kg) (mean ± SE)
1 CCCCCC	7	139.33 ± 3.09	150.67 ± 3.49	48.33 ± 2.18	467.33 ± 36.02	283.73 ± 19.78
2 CCCCCT	15	140.29 ± 2.03	152.29 ± 2.29	46.43 ± 1.43	515.14 ± 23.58	259.40 ± 12.95
3 CCCCTT	8	137.50 ± 3.79	153.00 ± 4.28	45.50 ± 2.68	496.00 ± 44.11	264.10 ± 24.22
4 CCCTCC	8	145.00 ± 3.79	152.50 ± 4.28	47.00 ± 2.68	524.00 ± 44.11	255.90 ± 24.22
5 CCCTCT	25	140.13 ± 1.38	151.40 ± 1.56	47.57 ± 0.98	490.80 ± 16.11	263.33 ± 8.85
6 CCCTTT	40	141.00 ± 1.05	150.81 ± 1.19	46.73 ± 0.74	498.31 ± 12.23	271.83 ± 6.72
7 CCTTCC	6	142.00 ± 3.79	152.00 ± 4.28	47.50 ± 2.68	503.50 ± 44.11	285.10 ± 24.22
8 CCTTCT	16	139.67 ± 1.79	153.33 ± 2.02	46.90 ± 1.26	490.11 ± 20.79	270.44 ± 11.42
9 CCTTTT	50	141.30 ± 0.88	151.68 ± 0.99	47.65 ± 0.66	501.70 ± 10.26	266.39 ± 5.63
10 GCCCCC	10	140.50 ± 2.68	147.75 ± 3.02	45.75 ± 1.89	463.50 ± 31.19	264.75 ± 17.13
11 GCCCCT	28	141.80 ± 1.20	153.95 ± 1.35	48.45 ± 0.85	503.25 ± 13.95	264.54 ± 7.66
12 GCCTCT	40	140.07 ± 1.03	151.78 ± 1.16	47.37 ± 0.73	520.41 ± 12.01	279.10 ± 6.59
13 GCCTTT	22	140.73 ± 1.62	150.09 ± 1.82	47.00 ± 1.14	485.82 ± 18.81	265.65 ± 10.33
14 GCTTCT	15	141.40 ± 1.70	152.40 ± 1.91	45.80 ± 1.20	512.60 ± 19.73	263.54 ± 10.83
15 GCTTTT	11	141.88 ± 1.90	150.75 ± 2.14	47.38 ± 1.34	511.25 ± 22.06	266.58 ± 12.11
16 GGCCCC	25	139.79 ± 1.30	149.18 ± 1.47	46.53 ± 0.91	483.76 ± 15.13	265.93 ± 8.31
17 GGCCCT	9	136.40 ± 2.40	148.20 ± 2.71	44.00 ± 1.69	479.20 ± 27.90	258.28 ± 15.32
18 GGCTCT	10	136.67 ± 2.19	146.00 ± 2.47	45.67 ± 1.54	480.83 ± 25.47	264.87 ± 13.99
19 GGTTCC	5	139.00 ± 3.79	151.50 ± 4.28	43.00 ± 2.68	509.00 ± 44.11	265.35 ± 24.22
Significance (*P* value)		0.83	0.59	0.75	0.93	0.99

*SE* standard error

## Discussion

In previous studies, the *MYH*_3_ gene is the majority myosin isoform in embryonic and neonatal muscle fibers and its expression declines after birth to become undetectable around 3 weeks postnatal [45]. *MYH*_*3*_ expression demonstrates in the early looping heart, and subsequently throughout the myocardium of the outflow tract, and at lower levels to the ventricular chamber [38]. Knockdown of *MYH*_*3*_ in the chick resulted in abnormal atrial septal development, similar to that seen upon *α*-*MYH* knockdown [46, 47]. These results support the idea that *MYH*_*3*_ is a fundamental process in skeletal muscle and heart development. Another study finds that myosin myopathies have evolved as a new group of muscle diseases caused by mutations in skeletal muscle myosin heavy-chain genes [48].

Up to now, there are few polymorphisms detected in the *MYH*_*3*_ gene, it may be related to meat quality traits. We choosed it as a candidate gene to identify its SNPs and analyze the associations between the polymorphism and growth and carcass traits in the Qinchuan cattle population, which would lay the foundation for the study on the function of *MYH*_*3*_ gene in the cattle breed.

The allelic and genotypic frequencies, *PIC, He, Ne* and *χ2* values for SNPs showed considerable variability in Qinchuan cattle, the allelic frequency of the T alleles in five loci were up to 0.42 and C alleles in five loci were up to 0.40. *PIC, He and Ne* of SNP3 were higher than other four SNPs, showed the high polymorphism, high genetic variability, high ability to maintain allelic stability during selection or mutation, so SNP3 was likely to be useful in breeding programs for Qinchuan cattle. Chi-squared tests of SNP1, SNP3, SNP4 and SNP5 did not agreed with Hardy–Weinberg equilibrium (0.01 < *P* < 0.05, *P* < 0.01, *P* < 0.01 and *P* < 0.01, respectively), while SNP2 met with the Hardy–Weinberg equilibrium (*P* > 0.05).

In this study, we first reported the novel five SNPs in bovine *MYH*_*3*_ gene, and its association analysis with the growth and carcass traits in bovine. The genotypes at locus C1878T had a significant effect on slaughter weight. The genotypes at locus T2010C had a significant effect on withers height and carcass weight. The genotypes at locus G3746C had a significant effect on withers height. The genotypes at locus C7315T had a significant effect on slaughter weight. Based on these study, the individuals with better performance with the CT genotype at locus C1878T and TT genotypes at locus T2010C and GG genotypes at locus G3746C and CC genotypes at locus C7315T could be used for the development of new breeds of beef cattle in China, and these four locus could be used as remarkable molecular markers for better performance in the bovine industry.

The result of haplotype analysis of three major loci showed that eight different haplotypes were identified which get nineteen combined haplotypes, and all nineteen combined genotypes were found in this study animal DNA samples, four major combined haplotypes accounting for 51.9 % of the haplotypes were obtained as follows, CCCTTT: (12.3 %), CCTTTT: (17.5 %), GCCTCT: (9.4 %), GCCCCT: (12.7 %). But the associated analysis suggested that no significant differences were detected between the combined genotypes of three SNPs and growth and carcass traits in Qinchuan cattle (*P* > 0.05). So this study indicated that combined haplotypes did not increase growth or carcass traits of Qinchuan cattle.

In our study, three mutations in the bovine *MYH*_*3*_ gene were found in the exon region, polymorphisms in these regions could alter gene expression. The SNP g. +7294C>T was a missense mutation Arg2432Cys. It is possible to change the important physiological function in secondary and tertiary configuration of *MYH*_*3*_ protein as well as affect the biological function of protein, and it needs to study further. Our study was the first report of five novel SNPs in the bovine *MYH*_*3*_ gene, and we analyzed their association with growth and carcass traits in Qinchuan cattle. The results showed that some genotypes had a significant effect on the growth and carcass traits. Therefore, these mutations of *MYH*_*3*_ gene might positively influence the growth and carcass traits in Qinchuan population. In conclusion, this study will be contributed to geneticists and breeders as a molecular marker for better performance in the bovine industry. While the further research is still needed to clarify the role on the genetic variants of the *MYH*_*3*_ gene, and to analyze the mRNA expression levels of the *MYH*_*3*_ gene. Hence, would be a candidate gene worthy of further investigation.

## Abbreviations

PCR-RFLP: Polymerase chain reaction-restriction fragment length polymorphism
SNPs: Single nucleotide polymorphisms
*MyHC*: Myosin heavy chain
*MYH*_*3*_: Myosin heavy chain 3
*HMM*: Heavy meromyosin
*LMM*: Light meromyosin
LD: Linkage disequilibrium
*He*: Heterozygosity
*Ho*: Homozygosity
*PIC*: Polymorphism information content
